# Thrombose de la veine porte au cours d'une hépatite auto immune type 1

**DOI:** 10.11604/pamj.2013.14.130.1435

**Published:** 2013-04-03

**Authors:** Bouomrani Salem, Farah Afef, Bouassida Nadia, Ayadi Nabil, Béji Maher

**Affiliations:** 1 Service de Médecine Interne, Hôpital Militaire de Gabes 6000-Tunisie; 2 Service de Radiologie, Hôpital Militaire de Gabes 6000-Tunisie

**Keywords:** Hépatite auto-immune, thrombose porte, Autoimmune hepatitis, portal vein thrombosis

## Abstract

Notre but est de rapporter une observation particulière de thrombose de la veine porte survenant au décours d'une hépatite auto-immune type 1 non compliquée qui, à notre connaissance, n'a pas été rapportée auparavant. Il s'agit d'une patiente de 35 ans connue ayant une dermatose bulleuse auto immune (DBAI) type pemphigus confirmée histologiquement et immunologiquement qui fut explorée pour des coliques hépatiques avec élévations des transaminases et un épisode d'ictère spontanément résolutif. Les explorations ont permis de retenir le diagnostic d'une hépatite auto immune de type 1. Traitée par corticothérapie systémique à la dose de 1 mg/kg/j pour sa DBAI, l'évolution était favorable avec stabilisation simultanée de l'atteinte hépatique durant 19 ans. On découvre sur l'échographie abdominale de contrôle une thrombose partielle du tronc porte confirmée par le scanner X abdominal. Le bilan étiologique de cette thrombose est resté négatif. De même il n'y avait pas de signes cliniques, biologiques, endoscopiques ou radiologiques de cirrhose ni de dégénérescence maligne. Elle était efficacement antigoagulée par les antagonistes de la vitamine K. Dans notre observation le bilan étiologique; aussi exhaustif que possible, de cette thrombose est resté négatif, éliminant en particulier une cirrhose, une dégénérescence maligne et un syndrome des anti phospholipides associé et permettant de la rattacher directement à l'hépatopathie chronique auto immune.

## Introduction

Les hépatites auto-immunes (HAI) sont des affections chroniques du foie d'origine dysimmunitaire. Leur diagnostic repose sur les critères du Groupe International des Hépatites Autoimmunes établies en 1993 et révisés en 1999 [1, 2]; des critères plus simplifiés ont été récemment proposés [3]. Parmi les HAI classables, celle de type 1 est la plus fréquente. Elle survient particulièrement chez l'enfant et la femme jeune [4]. Sur le plan immunologique, elle se caractérise par la présence d'auto anticorps antinucléaires et/ou anti anti muscles lisses de spécificité anti actine [4]. La survenue d'une thrombose de la veine porte (TVP) au cours du suivie évolutif d'une HAI doit faire rechercher principalement une greffe néoplasique sur foie de cirrhose ou une autre affection thrombogène associée. Nous rapportons une observation particulière de TVP survenant au décours d'une HAI simple non compliquée de cirrhose ni de cancer et en dehors de toute autres associations à risque thrombotique.

## Patient et observation

Patiente de 35 ans aux antécédents familiaux d'auto-immunité (fille lupique et sœur ayant une hépatopathie auto-immune) est connue ayant une dermatose bulleuse auto immune (DBAI) type pemphigus diagnostiquée en 1991 et confirmée par une biopsie cutanée avec immunofluorescence directe. Elle fut explorée pour des coliques hépatiques avec élévations des transaminases et un épisode d'ictère spontanément résolutif dans les antécédents (il y a trois ans). La biologie montrait une cytolyse hépatique avec ASAT à 8N et ALAT à 10N sans cholestase ni stigmates d'insuffisance hépato cellulaire. Elle ne rapportait pas de prises médicamenteuses ni d'alcoolisme.

**Figure 1:**
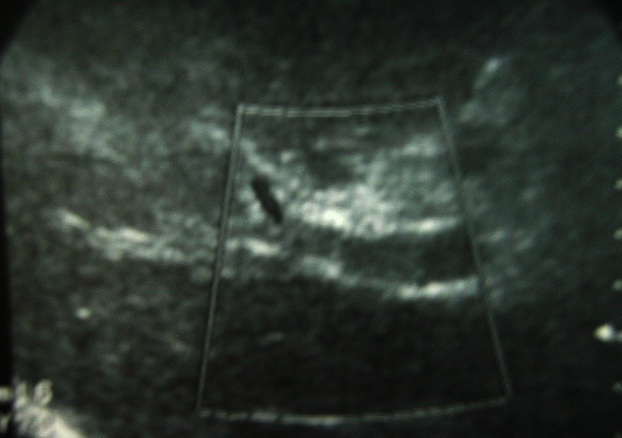
Thrombose de la veine porte en écho-doppler hépatique

**Figure 2:**
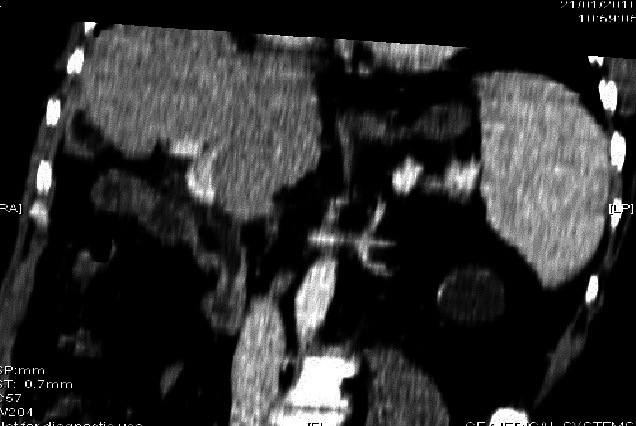
Scanner X abdominal injecté en reconstruction sagittale montrant la thrombose partielle et étendue du tronc porte.

Les sérologies des hépatites virales B et C étaient négatives. L'échographie hépatique montrait un foie dysmorphique d'hépatopathie chronique sans signes d'hypertension portale ni lésions focales suspectes. Les anticorps anti nucléaires étaient négatifs ainsi que les anti mitochondries type M2 et les anti LKM1. Les anticorps anti muscles lisses étaient positifs et à des taux significatifs. Le reste du bilan biologique était sans anomalies en dehors d'une hypergammaglobulinémie polyclonale à 19 g/l faite principalement d'IgG. Ainsi le diagnostic d'une hépatite auto immune de type 1 fut retenu (ALAT > 5N, IgG élevées et anti muscles lisses positifs). Traitée par corticothérapie systémique à la dose de 1 mg/kg/j pour sa DBAI, l'évolution était favorable avec stabilisation simultanée de l'atteinte hépatique durant 19 ans. Le traitement par azathioprine était refusé par la patiente. Actuellement elle est âgée de 56 ans et est asymptomatique avec des transaminases à 1,5N sans signes de cirrhose (pas de stigmates biologiques d'insuffisance hépato cellulaire, pas de varices gastriques à la fibroscopie). On découvre sur l'échographie abdominale de contrôle une thrombose partielle du tronc porte (Figure 1) confirmée par le scanner X (Figure 2). L'alpha-foetoprotéine était normale, il n'y avait pas de syndrome inflammatoire biologique, les explorations morphologiques thoraciques et abdomino-pelviennes (échographie abdominale, scanner X abdomino-pelvien et thoracique) ainsi qu'un scanner X cérébral n'ont pas objectivé de tumeurs et la numération formule sanguine était normale. Les marqueurs tumoraux: CA19-9, CA125, CA15-3, ACE, NSE (antigène neuronal spécifique) n'étaient pas élevés. L'IRM hépatique n'a pas montré de signes de dégénérescence. La bandelette urinaire était normale, de même que le HLM; le test de Hame d'Acier était négatif et l'étude cytogénétique n'a pas objectivé de clone HPN. Le myélogramme et le caryotype médullaires n'ont pas montré d'anomalies. La patiente ne présentait pas de signes cliniques pour une maladie de Behçet; l'examen oculaire ne montrait pas d'anomalies, le pathergy test n'objectivait pas d'hypersensibilité cutanée non spécifique et le typage HLA était négatif pour le B51. L'examen gynécologique était normal; complété par un frottis cervico-vaginal, une échographie pelvienne et une écho-mammographie qui n'ont pas objectivé d'anomalies. L'endoscopie digestive (coloscopie totale et gastroscopie) n'a pas objectivé de signes suspects de malignité.

Le doppler des membres inférieurs ne montrait pas de thromboses veineuses à ce niveau ni de signes d'insuffisance veineuse chronique. Le bilan de thrombophilies était négatif (TCA, AAN, anticorps anti cytoplasme des polynucléaires neutrophiles, cryoglobulines, anti phospholipides, anti thrombine III, facteur V de Leiden, proteines C et S). Par ailleurs aucune prise de médicaments susceptibles d'induire un état d'hypercoagulabilité sanguine ou favoriser la thrombogenèse n'a été signalée par la patiente.

## Discussion

La survenue d'une thrombose de la veine porte (TVP) chez un patient porteur d'une HAI peut résulter de deux mécanismes: 1) l'association à d'autres affections auto immunes caractérisées par un risque thrombogène élevé; en particulier un lupus érythémateux systémique ou un syndrome des anti phospholipides [5, 6]. Ces associations sont retrouvées dans 40-50% des HAI [6] et la présence des anticorps anti phospholipides reste la plus fréquente: en effet 17 cas/24 soit 70.8% des patients avec HAI dans le série de Branger et al. avaient des APL positifs avec quatre cas de syndrome primitif des APL défini soit 28.6% [6]. D'autre part les atteintes hépatiques, TVP comprise, restent les manifestations abdominales les plus fréquentes du SAPL primitif [7]; 2) l'installation d'une cirrhose avec ou sans dégénérescence cancéreuse [8]. Cette éventualité reste rare [9] puisque le pouvoir cirrhogène des HAI type 1 est faible comparativement à celui des HAI type 2 [4, 10]: Hakem et al. dans leur série de 50 cas d'HAI n'ont retrouvé que seulement trois greffes néoplasiques [10].

En dehors de ces deux situations, la TVP associée à une HAI n'a été rapportée qu'une seule fois chez une femme de 73 ans ayant une HAI type 1 compliqué d'une atrophie extrême du lobe droit du foie [11].

Pour notre patiente, le bilan étiologique; aussi exhaustif que possible, de cette thrombose est resté négatif, éliminant en particulier une cirrhose, une dégénérescence maligne et un syndrome des anti phospholipides associé et permettant de la rattacher directement à l'hépatopathie chronique auto immune.

La formation d'une telle thrombose pourrait être expliquée par les perturbations hémodynamiques locales du système porte, conséquence de l'hépatopathie chronique; en effet une étude récente a montré que la réduction du flux sanguin au niveau de la veine porte était le seul facteur prédictif du développement des TVP au cours des hépatopathies chroniques en analyse multi variée [12] suggérant ainsi un rôle plus important des facteurs hémodynamiques locaux que celui des déficits systémiques acquis en agents anticoagulants dans la pathogénie des TVP au cours des affections chroniques du foie [12].

## Conclusion

Notre observation, est à notre connaissance, la première illustrant la possibilité de voir se développer une TVP au cours d'une HAI simple en dehors de toute cirrhose, dégénérescence maligne ou autre maladie thrombogène associée.
